# Sensor-Based Pasture Quality Monitoring: Supporting Grazing Management and Preventing Nutritional and Metabolic Disorders in Ruminants

**DOI:** 10.3390/s26175472

**Published:** 2026-08-29

**Authors:** Henrique Pinto, Ricardo Santos, Guilherme Defalque, Francisco J. Moral, João Serrano

**Affiliations:** 1 MED—Mediterranean Institute for Agriculture, Environment and Development and CHANGE—Global Change and Sustainability Institute, University of Évora, Mitra, Ap. 94, 7006-554 Évora, Portugal; henrique.pinto@uevora.pt; 2 College of Computing, Federal University of Mato Grosso do Sul, Campo Grande 79070-900, Mato Grosso Do Sul, Brazil; ricardo.santos@ufms.br (R.S.); guilherme.defalque@ufms.br (G.D.); 3 Departamento de Expresión Gráfica, Escuela de Ingenierías Industriales, Universidad de Extremadura, Avenida de Elvas s/n, 06006 Badajoz, Spain; fjmoral@unex.es

**Keywords:** precision animal nutrition, biomass estimation, machine learning, sward heterogeneity, decision support systems, precision livestock farming

## Abstract

**Highlights:**

**What are the main findings?**

- Remote sensing technologies, including multispectral, hyperspectral, radar, and LiDAR, provide consistent, scalable measurements of pasture biomass, structure, and emerging indicators of forage quality.
- Advances in data fusion, spectral unmixing, and machine learning significantly improve the monitoring of heterogeneous swards and enhance the reliability of pasture quality assessment.

**What are the implications of the main findings?**

- Integrating remote sensing outputs into grazing decision support systems can improve allocation strategies, reduce nutritional risks, and support timely management interventions.
- Enhanced pasture monitoring contributes to more sustainable, health-focused livestock production by supporting better nutritional management and reducing the likelihood of metabolic disorders in ruminants.

**Abstract:**

Pasture quality monitoring is essential for optimizing grazing management and reducing the incidence of nutritional and metabolic disorders in ruminants, yet conventional field-based measurements remain labor-intensive and limited in spatial coverage. This review examines how remote sensing (RS) technologies can support pasture-based livestock systems by providing timely, scalable assessments of biomass, botanical composition, and nutritive attributes. Data from multispectral, hyperspectral, radio detection and ranging (RADAR), and light detection and ranging (LiDAR) sensors, acquired via satellite, unmanned aerial vehicle (UAV), and proximal platforms, are combined with machine learning (ML) methods and radiative transfer models to derive pasture biophysical and quality indicators. The reviewed evidence shows that RS reliably estimates pasture biomass and structural traits, while advances in spectral unmixing, data fusion, and artificial intelligence (AI) improve the characterization of heterogeneous swards and support emerging indicators related to forage quality. Integrating these remotely sensed metrics into grassland decision-support frameworks can enhance grazing allocation, inform fertilization and irrigation decisions, and help detect conditions associated with nutritional imbalances. Overall, the synthesis demonstrates that RS, particularly when combined with advanced modelling and cloud-based processing, offers a robust pathway for improving pasture monitoring and strengthening the nutritional management of ruminants, thereby supporting more sustainable and animal welfare-focused grazing systems.

## 1. Introduction

Grasslands play a vital role in terrestrial ecosystems, covering a significant portion of the Earth’s land surface. They provide essential forage for grazing animals and offer important ecosystem services such as storing carbon and supporting wildlife habitats, which help reduce the impacts of climate change and protect biodiversity. In well managed livestock systems, pastures remain the main source of feed for animals [1].

In recent decades, the use of pastureland in Europe has declined by about 30%, largely due to the higher efficiency and greater control over feed that indoor confined systems provide. In contrast, when climates are suitable, pasture-based systems can offer both economic and environmental advantages over indoor feeding. However, these systems face challenges because grasslands vary in space and time, making feed availability harder to control. The amount of grazeable herbage in a pasture can fluctuate widely due to selective grazing, the presence of dung patches, and seasonal changes in grass growth. This variability makes it difficult to accurately estimate and manage the feed available to grazing animals [2]. As a result, incorporating precision technologies has become essential for tracking environmental conditions and managing grazing animals more effectively. Precision livestock farming (PLF) is an emerging, multidisciplinary field that uses advanced technological tools to improve the overall management of farming systems, supporting more informed decisions regarding grazing practices and the land’s ability to sustain livestock [3]. Remote sensing (RS), alongside other technologies, has evolved into a vital tool across agriculture, earth science, and environmental studies, providing valuable information on shifts in land use, changes in climate, and the management of natural resources [4].

Cow–calf operations worldwide largely rely on natural grazing pastures, whose productivity and energy content are strongly influenced by climate. Maintaining optimal grazing intensity throughout the year ensures that forage remains in a young, productive stage, which maximizes both biomass yield and metabolizable energy (ME) availability. This approach not only provides a more consistent and high-quality energy source for the grazing herd but also supports the long-term ecological health and sustainability of the pasture ecosystem [5]. And to make decisions effectively, farmers require information on key biophysical features of pastures. This includes above-ground biomass, canopy cover, plant density, and height to assess pasture quantity, as well as species composition, palatability, digestibility, and nutritional attributes, such as nitrogen (N), crude protein (CP), neutral detergent fiber (NDF), and acid detergent fiber (ADF), to evaluate pasture quality [1]. This information can be crucial when aiming to prevent nutritional and metabolic disorders in grazing ruminants, improving animal health and performance.

According to Wu [6], metabolic disorders on farms often arise when animals experience overlapping nutrient imbalances, whether deficiencies or excesses, which can also make them more vulnerable to bacterial, viral, fungal, or parasitic infections. When normal metabolic processes are disrupted, a range of conditions may appear. Carbohydrate-related imbalances can lead to issues such as ruminal acidosis and bloat, while irregularities in lipid metabolism contribute to problems like bovine fatty liver syndrome, ketosis in high-producing dairy cows, low-fat milk syndrome, and pregnancy toxemia. These disorders pose a significant challenge in cattle production, impacting both animal welfare and farm productivity. Some conditions, such as jejunal hemorrhage syndrome, are even life-threatening and could potentially be prevented through continuous pasture quality monitoring. Monitoring CP levels relative to DM can support timely nutritional interventions, helping to avoid excessive protein intake and reduce the risk of digestive and metabolic disorders. But effectively preventing and treating these conditions requires a clear understanding of their causes and management options [7].

Current modeling approaches that utilize satellite observations include parametric, nonparametric, and physically based frameworks. Parametric models—often reliant on vegetation indices—are computationally efficient but may be constrained by factors such as dense canopy structure or soil reflectance effects. In contrast, nonparametric models, including machine learning (ML) techniques such as random forest (RF) and support vector regression, offer greater adaptability and improved performance for estimating forage biomass and nutritional attributes. Physically based approaches, such as canopy radiative transfer models, further enhance predictive capability by simulating vegetation processes directly from satellite-derived inputs. These RS methodologies enable near real-time evaluation of rangeland and grassland conditions, facilitating dynamic grazing management decisions, including stocking rate adjustments and forecasts of animal performance. Increasing evidence shows that integrating satellite data with field measurements and mechanistic models can optimize forage allocation, enhance livestock productivity, and advance the long-term sustainability of grazing systems [8].

**Figure 1:**
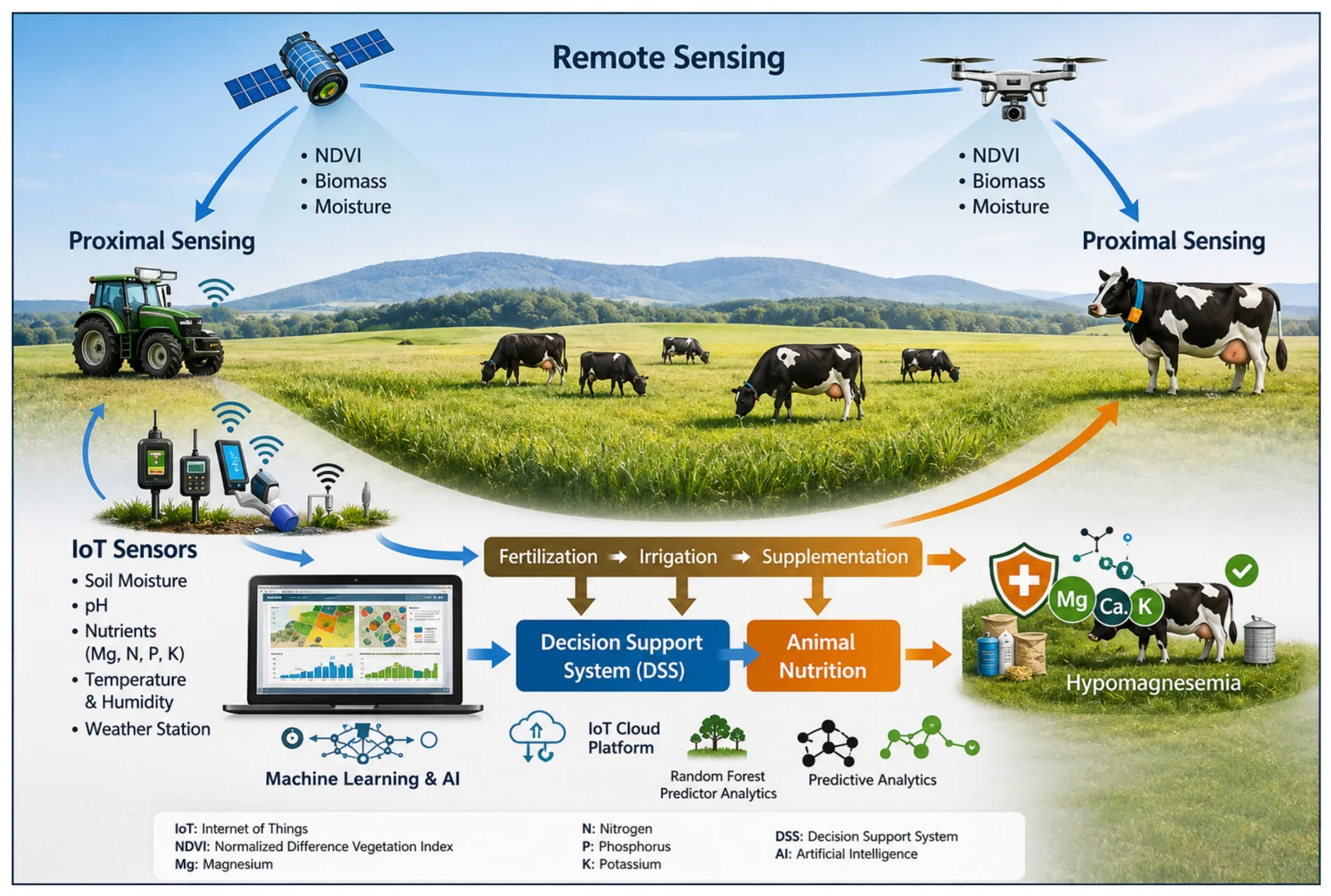
Conceptual overview of the integration of RS, PS, IoT, and DSS for pasture management and animal nutrition to prevent metabolic disorders in grazing livestock. Conceptual illustration developed by the authors.

This review is guided by the following research question: To what extent can remote, proximal, and animal-based sensing technologies improve pasture quality assessment and support grazing management decisions that may contribute to reducing nutritional risk factors associated with metabolic disorders in grazing ruminants (Figure 1).

The main objective of this review is to critically synthesize recent advances in RS, sensor technologies, and decision support systems (DSSs) applied to pasture-based livestock systems, with particular emphasis on pasture quality assessment, sward heterogeneity, and supplementation strategies. In addition, this review aims to identify current limitations and research gaps related to sensor calibration, data integration, and practical implementation, highlighting future research directions to enhance sustainable grazing management, precision nutrition, and animal health management.

To address this hypothesis, this review is structured to progressively connect pasture biophysical attributes with their nutritional implications and the technological pathways available to monitor them. Section 3 defines the key parameters of pasture quantity and quality that underpin nutritional management, while Section 4 critically evaluates how remote and proximal sensing (PS) technologies estimate these indicators and where their limitations lie. Section 5 establishes the physiological link between pasture attributes and rumen function, providing the biological basis for understanding how imbalances in dry matter, crude protein and fiber fractions contribute to metabolic disorders, which are examined in Section 6. Section 7 and Section 8 synthesize recent advances in machine learning (ML), IoT architectures and DSSs that integrate multisource sensor data to support grazing decisions. Finally, Section 9 and Section 10 consolidate the main limitations identified in the literature and outline specific research priorities needed to operationalize integrated sensing frameworks capable of improving pasture monitoring and reducing metabolic risks in grazing ruminants.

The novelty of this review lies in its interdisciplinary approach, integrating advances in RS, PS, AI, and DSSs with pasture nutritional assessment and ruminant health. Rather than reviewing sensing technologies in isolation, this work examines how these complementary tools can support precision grazing management and reduce the risk of nutrition-related metabolic disorders, while identifying current limitations and future research priorities for on-farm implementation.

## 2. Review Methodology

### 2.1. Review Design

This study was conducted as a structured narrative review designed to critically evaluate recent advances in sensing technologies for pasture quality monitoring and their application to nutritional management in grazing ruminants. Given the multidisciplinary nature of the topic, encompassing pasture science, RS, PS, animal nutrition, PLF, AI, and DSSs, a structured review approach was adopted to ensure comprehensive coverage while allowing critical synthesis across different research domains.

### 2.2. Literature Search Strategy

The literature search for this review was conducted using Google Scholar as the primary search engine, complemented by targeted searches in major scientific databases and publishers, including Scopus-indexed journals, NCBI (PubMed Central), ScienceDirect, Elsevier, IEEE Xplore, and MDPI. The search focused on peer-reviewed studies published between 2021 and 2025. This time window was selected because it corresponds to the period in which remote and proximal sensing technologies, UAV-based imaging, and ML applications for pasture monitoring became widely adopted in the scientific literature, ensuring that the review captures the most recent and operationally relevant developments.

Search strings were applied to the title, abstract, and keyword fields, combining terms related to pasture and grassland monitoring with sensing and analytical approaches, such as (“pasture quality” OR “forage quality” OR “grassland monitoring”) AND (“remote sensing” OR “proximal sensing” OR “NDVI” OR “spectroscopy” OR “sensor technologies”), as well as (“precision livestock farming” OR “decision support systems”) AND (“animal nutrition” OR “metabolic disorders”). Minor variations in these strings were used depending on database-specific search rules.

**Figure 2:**
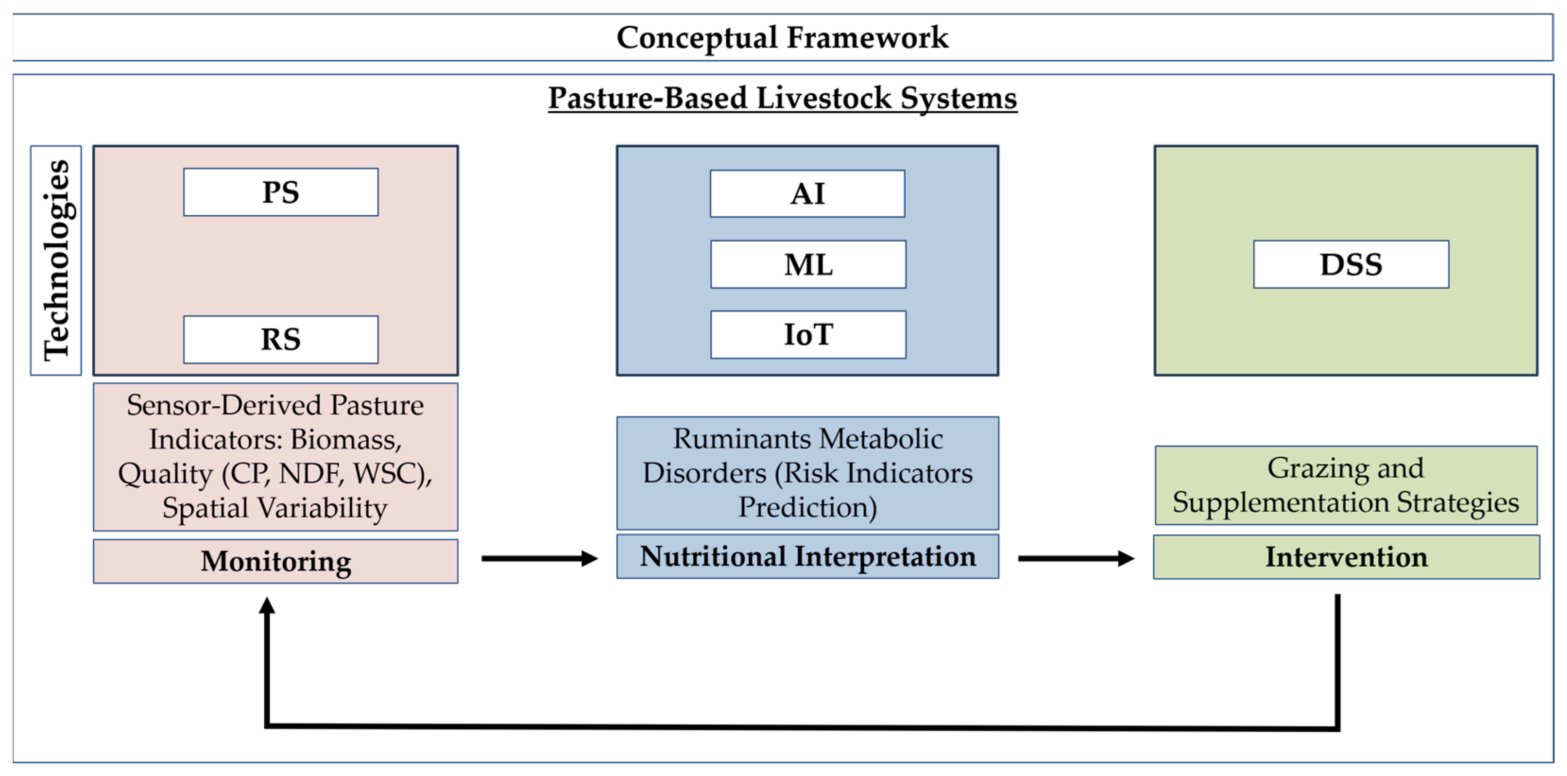
Conceptual framework of the integration of sensing technologies and decision support systems for pasture quality monitoring and grazing management.

The review process followed an iterative, narrative approach, with searches refined over time and complemented by backward citation tracking from the reference lists of selected articles. Although this review was conducted as a narrative review rather than a formal systematic review, the literature search and study selection followed a structured and transparent workflow to improve reproducibility. Articles were included based on their contribution to the themes of sensing technologies, pasture quality assessment, animal nutrition, and decision-support frameworks. To provide a conceptual overview of the topics addressed throughout this review, Figure 2 summarizes the proposed framework linking remote and proximal sensing technologies with AI, ML, IoT, and DSSs. This framework illustrates how sensor-derived information can support pasture quality assessment, nutritional interpretation, and management decisions aimed at optimizing grazing strategies and reducing the risk of nutrition-related metabolic disorders in grazing livestock.

**Figure 3:**
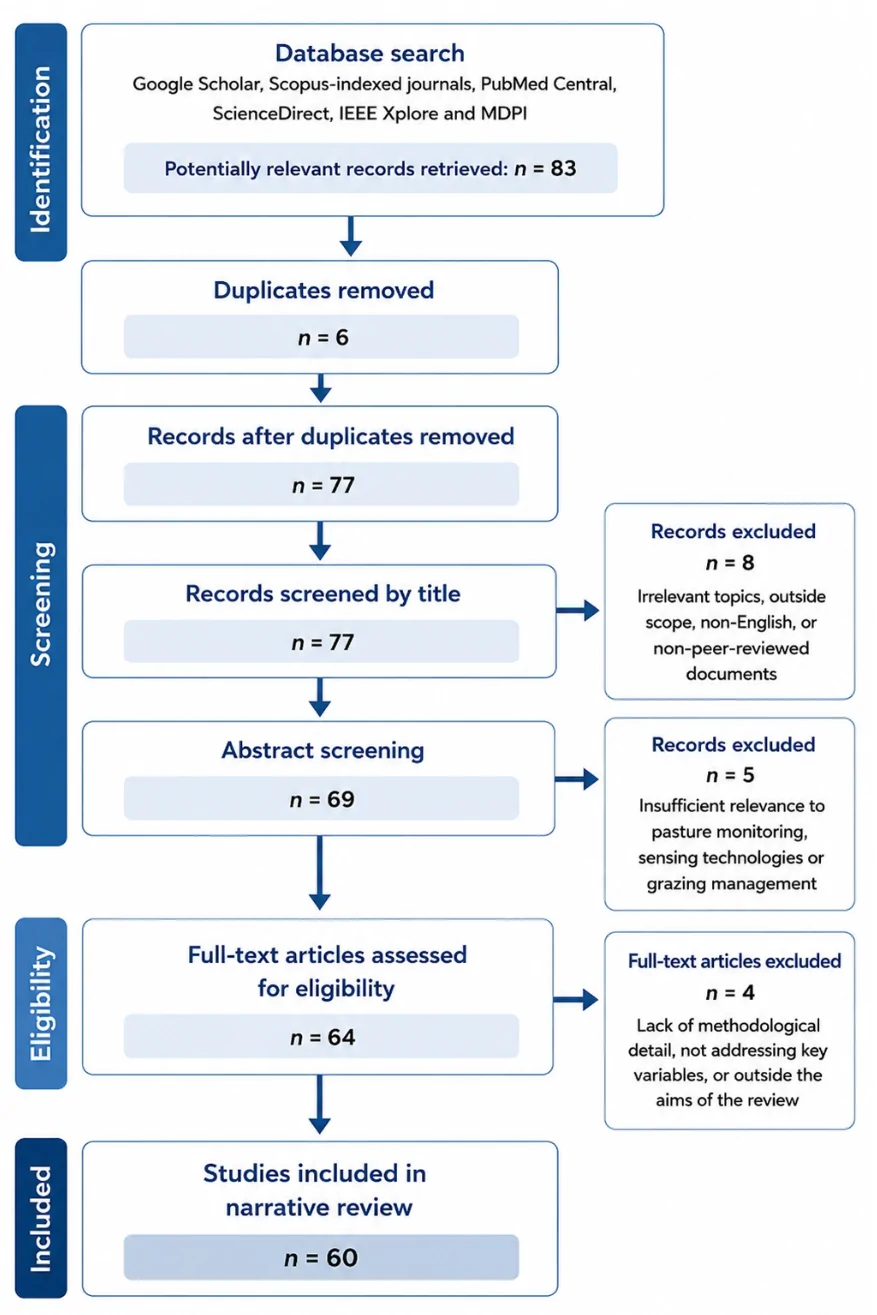
Flow diagram summarizing the structured literature search and study selection process adopted for this narrative review.

The screening process of the studies included duplicate removal, title and abstract screening, full-text eligibility assessment, and critical evaluation of scientific relevance. This workflow is summarized in Figure 3.

Studies were screened for relevance to the scope of the review, focusing on peer-reviewed articles written in English and published in leading journals within the fields of remote sensing, agronomy, precision agriculture (PA), sensors, and animal science. These included *Remote Sensing*, *Agronomy*, *Sensors*, and *Agriculture*, all of which are represented in the final reference list.

Studies were included if they fulfilled one or more of the following criteria:

- Peer-reviewed journal article;
- Pasture monitoring;
- Grazing livestock;
- Remote sensing;
- Proximal sensing;
- UAV;
- Satellite imagery;
- Hyperspectral sensing;
- LiDAR;
- Machine learning;
- IoT;
- DSS.

Publications were excluded if they:

- Focused exclusively on housed livestock systems;
- Addressed crop production without pasture applications;
- Consisted only of conference abstracts or editorials;
- Lacked methodological information;
- Duplicated previous publications.

Although the primary literature search focused on studies published during the last five years, several earlier publications were intentionally retained because they constitute foundational references in pasture ecology, rumen physiology, remote sensing methodology, and decision support systems. These seminal contributions remain essential for understanding the evolution of current sensing technologies and their application to grazing systems.

Rather than simply summarizing published studies, the selected literature was critically evaluated according to several technological and operational criteria, including measurement capability, calibration requirements, robustness, scalability, operational cost, transferability between production systems, and suitability for integration into precision livestock farming decision-support systems.

Several limitations should be acknowledged. First, despite adopting a structured search strategy, this study remains a narrative review and therefore does not attempt quantitative meta-analysis. Second, only English-language peer-reviewed publications were considered. Third, rapid technological developments may lead to the appearance of new sensing platforms shortly after publication. Finally, differences in pasture species, climatic conditions, calibration procedures, sensor configurations, and validation metrics limit direct quantitative comparison among published studies.

## 3. Pasture Availability and Quality Parameters

Pasture quality is a multidimensional concept encompassing structural, botanical, biochemical, and functional characteristics of the sward that directly influence nutrient availability, intake, digestibility, animal performance, and grazing behavior. In this context, forage quality refers more specifically to the nutritional and chemical characteristics of the plant material consumed by grazing animals. Thus, forage quality can be considered a component of the broader concept of pasture quality, rather than a synonymous term. From the perspective of PLF, the relevance of each pasture attribute depends not only on its nutritional significance but also on the feasibility of estimating it accurately using sensing technologies. Consequently, the practical value of pasture monitoring systems is determined by their capability to quantify variables that are simultaneously nutritionally relevant, spatially variable, and operationally measurable.

Pasture quantity, or availability (plant biomass/above-ground biomass, canopy coverage, density, and height [1]) can be assessed using metrics such as dry matter yield or fodder units [9], being commonly expressed as DM yield per unit area (DM, kg ha^−1^) [10]. But unlike monoculture crops (or those grown in rotation), pastures are complex natural ecosystems composed of multiple plant species, each of which may be grazed at different times, making pasture quality a more complex parameter. It encompasses both the characteristics of the sward and the nutritional quality of the forage available to grazing animals. The latter, referred to here as forage quality, is typically evaluated through indicators related to nutrient composition and digestibility of plant material [9]. From a sensing perspective, these parameters differ markedly in how detectable they are through remote or proximal technologies. Structural traits such as biomass, height and canopy cover tend to be more reliably estimated using multispectral indices or radar-derived metrics, whereas chemical traits like CP, NDF and water-soluble carbohydrates (WSC) require sensors with higher spectral resolution. This distinction is essential because it determines which technologies are realistically capable of supporting pasture monitoring on a scale.

In contrast, biochemical attributes remain considerably more difficult to estimate because their spectral signatures are often indirect, highly influenced by moisture content, plant phenology, species composition, and environmental variability. Most prediction models therefore require extensive local calibration and their transferability between production systems remains limited. But as mentioned above, the principal parameters routinely used for the assessment and reporting of pasture feed quality include CP (or N concentration), fiber fractions—specifically ADF and NDF—together with metabolizable energy (ME), organic matter digestibility (OMD) and WSC [2]. In addition to these core indicators, a broad range of other macro- and micro-nutrient constituents may be determined, depending on the pasture type and the characteristics of the production system [11]. Among all pasture quality parameters, mineral composition remains one of the least mature applications of RS. Essential elements such as calcium, phosphorus, magnesium, sodium, copper, selenium, and zinc generally lack distinctive spectral features that can be directly detected under field conditions. Consequently, mineral assessment currently relies predominantly on indirect prediction models or complementary laboratory analyses.

Other parameters include pasture moisture content (PMC), which reflects the nutritional value, fiber content, and water status of the vegetation, respectively, and vegetative vigor, often estimated using the Normalized Difference Vegetation Index (NDVI), which provides a non-destructive indicator of chlorophyll content and overall pasture health [10,12]. Although NDVI and similar indices are widely used, their sensitivity to biochemical traits is limited, especially under dense canopies where spectral saturation occurs. This highlights the need for complementary sensing approaches when the goal is to estimate quality-related parameters rather than only structural ones.

In recent research, a novel Pasture Quality Index (PQI) was developed by Adar et al. [9] to integrate multiple forage nutritional attributes into a single metric and enable large-scale pasture quality assessment. The PQI is calibrated using field measurements of pasture composition and nutritional parameters, which are then linked to spectral information derived from high-resolution RS data. By modeling relationships between pasture quality and vegetation spectral signatures, the PQI can be spatially and temporally predicted using satellite imagery [9]. The development of composite indices such as PQI illustrates a broader trend: individual nutritional parameters are often difficult to estimate in isolation, but their combined spectral expression can be more robustly captured through multivariate or ML models. This reinforces the importance of integrating multiple sensing modalities, a theme further explored in Section 4.

Nevertheless, CP and NDF are still the most widely applied parameters for which RS studies show the greatest variability in predictive accuracy. While biomass estimation often achieves R^2^ values above 0.70, CP and NDF typically show lower and more inconsistent performance, reflecting their biochemical complexity and the influence of phenological stage. This variability underscores the need for sensor fusion and advanced modelling approaches to improve reliability. But considering, on most occasions, that high-quality pastures should have higher CP levels (greater than 20% DM), and lower values of fiber (NDF; approximately 40% of DM) [10], depending on factors such as the species and/or breed of grazing animals or production status (dry period/lactation phase), the correct proportion of these two parameters may vary [13].

Table 1 presents a synthesis of the grass and forage quality indicators employed in recent studies for pre-grazing quality assessment.

From a sustainable and economical perspective, increased N utilization efficiency by cattle has a positive correlation not only with greater economical return, but also with decreased N losses to the environment (nitrous oxide, nitrates and ammonia) [20], avoiding atmospheric emissions and contamination of both surface and groundwater [21].

It has already been demonstrated that several animal and dietary factors affect N output [22,23], but dietary N concentration and intake show greater correlation with N output in manure in dairy cows [21]. In the light of this, 69% of inquired nutritionists in the United States of America reported a lower CP content in their nutritional formulations, in the last 3–5 years [24]. Regarding grass-based systems, it is known that grazing dairy cattle have a less efficient dietary N utilization, which can be mitigated by combining full-time grazing with in-barn supplementation of fresh forage and hay, thereby supporting low-input dairy operations while keeping nitrogen losses at moderate levels [25].

These nutritional dynamics further justify the need for accurate monitoring of CP, NDF and DM through sensing technologies, as small deviations in these parameters can have disproportionate effects on nitrogen use efficiency and metabolic stability. Therefore, understanding which parameters are most critical—and how well each sensing technology can estimate them—provides the analytical foundation for the technological assessment presented in Section 4.

## 4. Technologies Available for Pasture Monitoring

Effective grassland management relies on the availability of accurate, timely information on pasture status to support informed decision-making. Precise herbage allocation to livestock is a key component of efficient pasture management, as oversupply of pasture can result in biomass losses and a decline in sward quality, whereas insufficient herbage availability restricts animal intake and leads to reduced milk and meat production. This makes regular and reliable assessment of pasture biomass and nutritive quality a fundamental way for maximizing pasture utilization and productivity in grazing-based farming systems [2], achieving an increase of up to 15% in farm profitability [26]. However, the reliability of these assessments depends strongly on the sensing technology used, as available sensors vary widely in their ability to capture structural versus biochemical traits. This means that technology choice directly influences the accuracy of biomass estimation, CP prediction, or NDF assessment, and therefore the quality of grazing decisions.

In recent years, numerous studies have demonstrated the application of emerging technologies and methodologies for the assessment of pasture quality. Among the most successful approaches are those based on vegetation indices, which are derived from measurements of plant reflectance in different spectral regions. These indices can be rapidly obtained over large spatial extents using RS and, to a lesser extent, PS technologies, enabling efficient monitoring of pasture conditions and variability [27]. Despite their operational simplicity, VIs such as NDVI tend to saturate at moderate-to-high biomass levels and show limited sensitivity to biochemical traits like CP or NDF. This limitation reinforces the need for complementary sensing approaches, particularly when the objective extends beyond structural assessment to include nutritional quality.

RS encompasses all sensing techniques that operate at distances greater than two meters above ground level [28]. This category includes methodologies based on UAVs, manned aircraft, and satellite platforms. Over the past decade, research on the application of RS for predicting grass yield and quality has expanded substantially, driven by its capacity to monitor large areas with minimal labor input [2]. Satellite-based optical sensors, such as those onboard the Sentinel-2 mission, are widely used to monitor grassland canopy properties and derive vegetation indices based on spectral reflectance [29]. In addition to optical data, synthetic aperture radar (SAR) sensors provide complementary information on pasture structure and height, particularly under cloudy conditions. Optical sensors excel at capturing chlorophyll-related signals and canopy vigor, but they struggle with structural complexity and cloud cover. SAR, by contrast, penetrates clouds and provides structural information, yet its sensitivity to moisture and surface roughness can introduce noise. These complementary strengths explain why multisensor fusion (optical + SAR) consistently outperforms single-sensor approaches in biomass estimation.

UAVs equipped with multispectral or hyperspectral sensors have been extensively applied to estimate fresh and dry biomass, nitrogen content, and quality-related traits of grasslands with high spatial resolution. LiDAR systems further contribute by accurately characterizing the horizontal and vertical structure of vegetation, while aerial photogrammetry offers an effective alternative for three-dimensional pasture reconstruction and biomass estimation [29]. UAV-based hyperspectral systems are currently the most powerful tools for predicting CP, NDF and other biochemical traits, often achieving higher accuracy than satellite platforms due to their finer spectral and spatial resolution. LiDAR, although blind to chemical composition, provides unmatched structural detail, making it ideal for biomass modelling when combined with spectral data. This highlights a recurring pattern: no single technology captures all relevant pasture attributes, and integrated approaches consistently yield superior results.

A substantial body of work has also focused on machine-mounted sensing systems integrated into harvesting and mowing equipment, including load cells, capacitance-controlled oscillators, mass-flow sensors, pendulum sensors, and forage-throughput sensors. These systems enable continuous, on-the-go measurement of biomass yield, moisture content, and material flow [29]. Machine-mounted sensors offer high accuracy at field scale but lack the spatial coverage of RS. Their greatest value emerges when used for calibration or validation of satellite and UAV models, reducing uncertainty and improving generalizability across heterogeneous swards. Additional proximal approaches include bale-weighing systems combined with GPS for spatial yield mapping, constant weighing methods on windrowers, curved plate sensors on mowing machines, and torque, pressure, or spring-force sensors mounted on windrowing devices and silage choppers.

Beyond machine-mounted systems, handheld and walking-based PS tools remain widely used for routine pasture assessment at farm level. The rising plate meter (RPM) is one of the most established non-destructive tools for pasture measurement, particularly in temperate grazing systems. The RPM estimates herbage mass based on compressed sward height, which integrates pasture height and density through the interaction between a weighted plate and the sward canopy. While its simplicity and compatibility with decision-support tools have contributed to its widespread adoption, measurement accuracy can be influenced by sward heterogeneity and variation in pasture structure [2]. RPM remains operationally attractive but its accuracy declines in heterogeneous or clumpy swards, making it less reliable in mixed-species pastures. This limitation is particularly relevant in Mediterranean and tropical systems, where structural variability is high.

Capacitance-based probes, such as Grassmaster^®^ II, represent an alternative PS approach for estimating pasture biomass. This technology relies on changes in an electrical signal generated by the surrounding vegetation and has been evaluated under a range of pasture conditions. Studies by Serrano et al. [30] demonstrated that Grassmaster^®^ II can provide in situ estimates of green and dry matter yield, although its performance is strongly affected by pasture and soil moisture conditions, necessitating site-specific or dynamic calibration models. Comparative analyses further indicate that, while operationally useful, the accuracy of capacitance probes is generally lower than that of the RPM, highlighting the importance of calibration and environmental context when applying proximal pasture measurement technologies. Together, RPM and capacitance probes illustrate a broader principle: proximal sensors offer high-frequency, low-cost measurements, but their performance is highly context-dependent. Their greatest value emerges when used to calibrate or validate RS models, reducing bias and improving transferability across sites.

**Figure 4:**
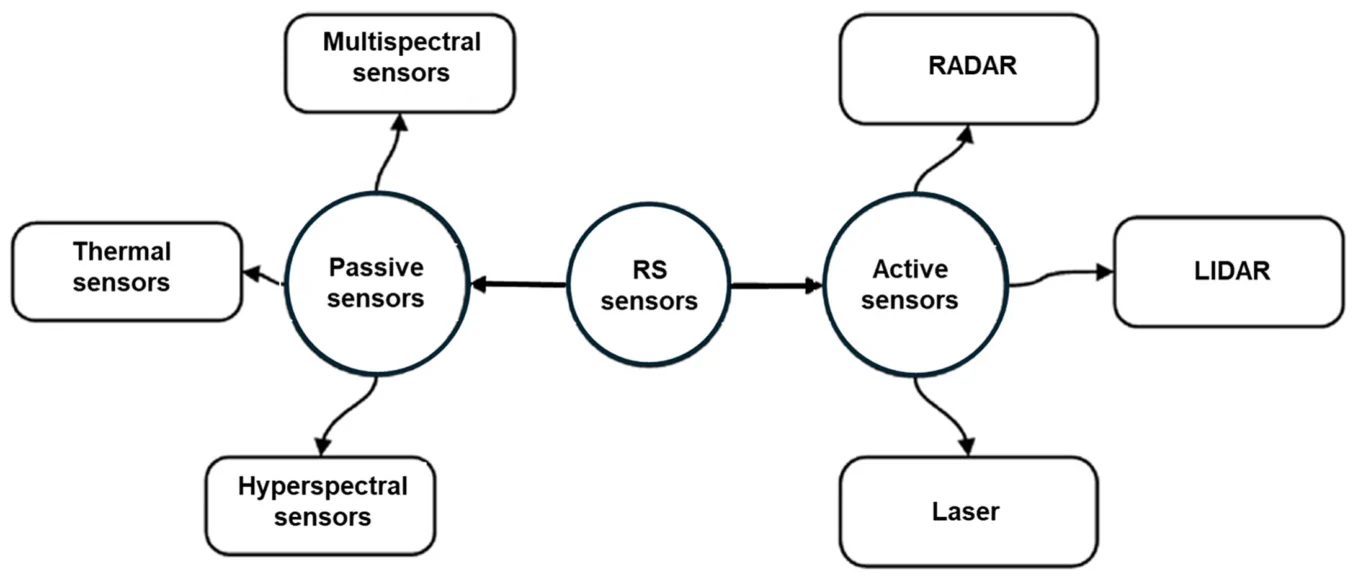
The typical classification of RS sensors used in pasture monitoring [1].

These PS approaches complement RS methodologies, commonly classified according to sensor type as either passive or active systems (multispectral, hyperspectral, thermal, radar, and LiDAR sensors, as illustrated in Figure 4).

Collectively, PS provides high-accuracy, operational measurements, enabling comprehensive and scalable pasture monitoring frameworks for improved grassland management and productivity [29]. Current evidence increasingly indicates that no individual sensing technology is capable of accurately estimating all relevant pasture characteristics under operational conditions. Consequently, recent research has progressively shifted towards multisensor fusion strategies combining satellite imagery, UAV observations, PS, LiDAR, meteorological information, soil measurements, and ML algorithms. Sensor fusion improves robustness by exploiting the complementary strengths of individual sensing platforms while reducing uncertainties associated with environmental variability and sensor-specific limitations.

Because pasture quality indicators differ considerably in their detectability, calibration requirements, and operational applicability, Table 2 summarizes the current capabilities of remote and proximal sensing technologies for estimating the principal structural and biochemical pasture parameters. To improve transparency and consistency, the qualitative assessments in Table 2 were assigned using a three-level semi-quantitative scale based on the evidence reported in the reviewed literature. A score of 1 (✓) indicates very low/limited performance or applicability, whereas a score of 3 (✓✓✓) indicates very high performance or applicability. For validation requirements, Low means that validation can generally rely on established measurements and relatively stable relationships; Moderate indicates that validation across environmental or pasture conditions is required; High indicates that extensive field validation and site-specific calibration are required because performance is strongly context-dependent. For operational readiness, Low indicates that technology or estimation approach is mainly experimental or requires substantial site-specific calibration; Moderate indicates that it is demonstrated under field conditions, but transferability and/or calibration requirements remain important; High indicates that it is demonstrated under operational field conditions with relatively mature workflows and established practical applications. In all cases, these ratings are intended as an evidence-based synthesis rather than as statistical estimates of sensor performance.

To complement the individual technology descriptions presented throughout this section, Table 3 provides a comparative assessment of the principal sensing platforms according to their capability for estimating biomass, CP, and NDF, together with their relative cost-effectiveness, calibration requirements, and operational applicability. This synthesis illustrates the trade-offs between sensing platforms and highlights that technology selection should be guided by the target pasture parameter, required accuracy, and practical implementation constraints rather than by a single performance criterion. Ratings were assigned using a five-level semi-quantitative scale based on the evidence synthesized from the reviewed literature. As previously indicated, the ratings represent an evidence-based synthesis of reported technological characteristics and field applications and should not be interpreted as pooled statistical performance metrics.

To minimize subjectivity, ratings were assigned using the following evidence-oriented criteria: (i) consistency of performance reported across studies, (ii) demonstrated field applicability, (iii) dependence on site-specific calibration, (iv) transferability across pasture conditions, and (v) technological maturity. Where quantitative comparisons were not possible because of differences in experimental design and performance metrics, the rating reflects the overall consistency and strength of the available evidence rather than a numerical average.

## 5. Pasture Parameters and Ruminal Activity in Grazing Ruminants

Ruminants possess a specialized digestive system, that besides mechanical and chemical processing, also relies on a symbiotic relationship between the host animal and rumen microorganisms, which enables the efficient conversion of fibrous plant materials into metabolizable nutrients through microbial fermentation [31]. This system comprises multiple specialized anatomical compartments and coordinated physiological processes that enable efficient digestion and nutrient absorption [32] and allows ruminants to utilize pasture resources that are largely inaccessible to non-ruminant species [31]. During the digestion process, saliva provides buffering capacity to maintain rumen pH, and the muscular esophagus enables both forward and reverse movement of ingesta, facilitating regurgitation and rumination. This process facilitates mechanical breakdown of food, increasing surface area for microbial action [32]. At the core of digestion is ruminal microbial fermentation, where bacteria, protozoa, and fungi break down complex carbohydrates (like cellulose) into volatile fatty acids (VFA), which supply most of the ruminant’s energy. Effective rumination and salivary buffering are essential to optimize this microbial activity, supporting nutrient absorption and overall health [32]. These specialized digestive characteristics also make ruminants particularly vulnerable to nutritional, metabolic, and digestive imbalances and disorders when feeding management is poor, emphasizing the need for careful dietary monitoring. From a pasture-monitoring perspective, this means that variations in all parameters that can be estimated to varying degrees by remote and proximal sensing have direct physiological consequences in the rumen. Therefore, the ability to monitor these parameters in real time is not merely agronomic; it is central to maintaining rumen stability.

This leads us to the role that variations in dietary nutritional levels can have on changes in rumen bacterial communities. The roughage source included in the diet can modify both the rumen microbiota and the associated carbohydrate-active enzyme profiles [33], since the nutrient composition of the feeds also correlated with that change [34]. Hao et al. [35] demonstrated that the fluctuations in dietary composition in CP, NDF, ether extract (EE) and starch can contribute to 32.49% of ruminal bacteria variation, correlating the rumen bacterial population and microbiota functions with rumen fermentation ability, which makes nutritional management a tool for regulating ruminal population [35]. These findings reinforce the importance of accurately characterizing pasture quality, as shifts in CP, NDF, WSC or DM translate into measurable changes in microbial populations and fermentation dynamics. In practical terms, sensing technologies capable of detecting early deviations in these parameters can serve as indirect indicators of rumen stability or impending dysfunction. For example, low DM and high WSC pastures (common in early vegetative stages) increase the risk of rapid fermentation and pH decline, while high NDF and low CP pastures (typical of mature swards) reduce intake and slow rumen turnover. These dynamics illustrate why pasture phenology is a critical determinant of rumen function and why real-time monitoring of these parameters is essential for anticipating metabolic imbalances.

**Figure 5:**
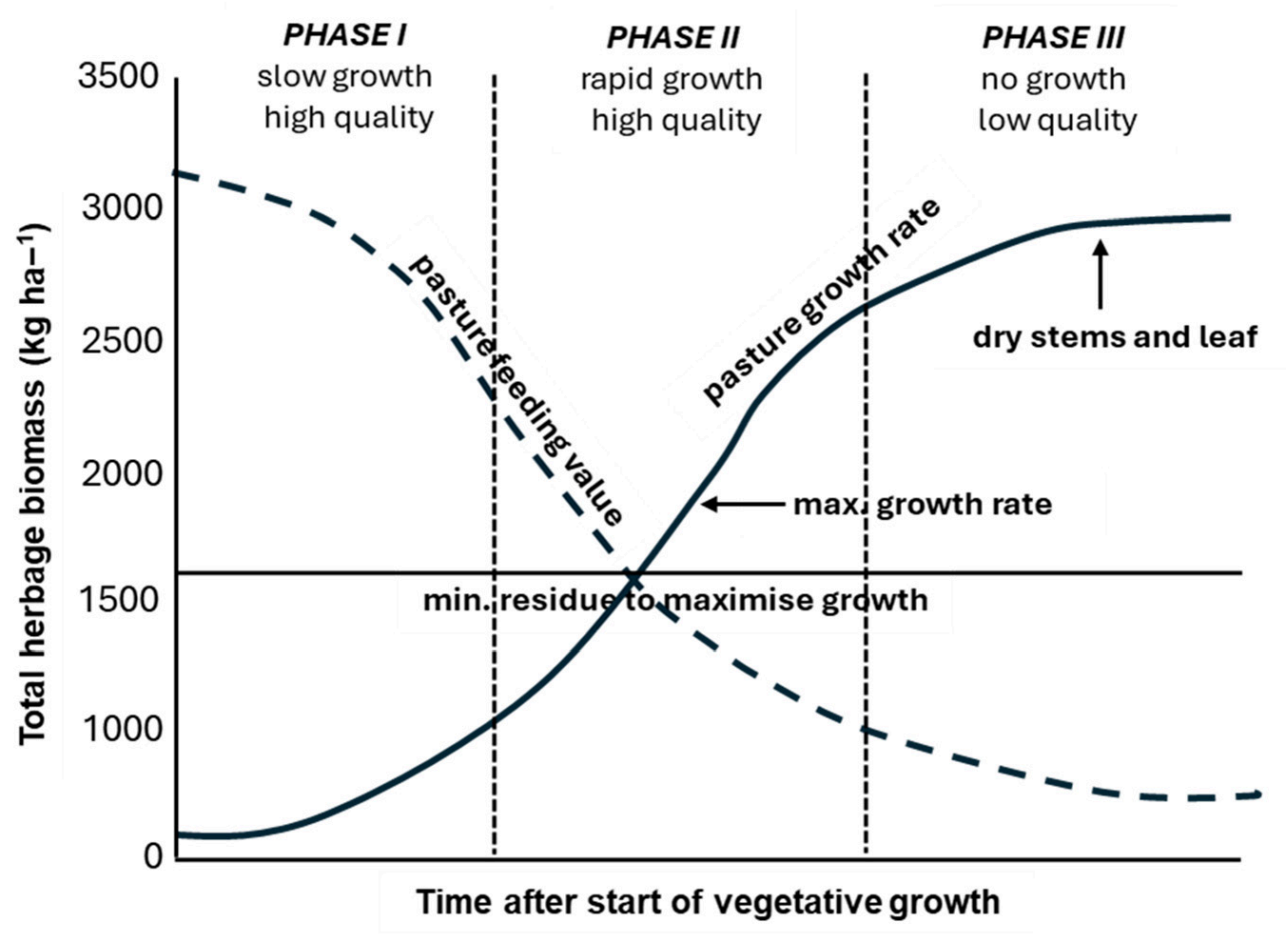
Growth curve of pastures over the growing season showing the three phases of growth and the changes in feeding value as the plants transition through each phase [36].

As explained in Figure 5 [36], from a nutritional standpoint, grasslands exhibit considerable variation in both their chemical composition and fermentative properties [37], due to spatial variability of soil and pasture characteristics and the pronounced temporal dynamics of grass species [38]. In addition, other factors such as environment, pasture conditions, stakeholders’ requirements, and herd behavior can also impact the nutrient demands of cattle, and that is when supplementation shows up as a tool for herd performance maintenance and improvement; however, performing the correct supplementation process can be tricky, especially when choosing the correct dosage and characteristics of the supplementation given [39]. Through PA technologies for pasture monitoring, like RS and PS, it is now possible to build supplementation DSSs, enabling a more precise nutritional management of ruminants in grasslands, based on real time data, and, as rumen responds rapidly to changes in pasture composition, and many of the parameters that drive these responses are precisely those that remote and proximal sensing technologies can monitor, it is possible to understand the mechanistic link between pasture quality sensing and metabolic disorder prevention in grazing ruminants.

## 6. Nutritional and Metabolic Disorders in Ruminants

Animal metabolism is controlled through a complex interplay of hormonal regulation, redox mediated signaling, protein covalent modifications, dietary inputs, environmental influences, and the intracellular availability of substrates and metabolites. These regulatory mechanisms collectively underpin essential biological functions, including growth, development, immune competence, productivity, overall health, and survival. Through coordinated short- and long-term metabolic adjustments, animals can respond effectively to nutritional, physiological, and environmental challenges, ensuring the integration of biochemical reactions, metabolic pathways, and acid–base regulation required to preserve physiological homeostasis [6]. Sustained disruption of physiological homeostasis can lead to the onset of disease, broadly described as an alteration in normal body structure or function that presents identifiable clinical signs and symptoms. In animal systems, metabolic disorders arise from a wide range of nutritional and metabolic disturbances, including poor dietary quality, insufficient or excessive nutrient intake, and failures in nutrient digestion, absorption, utilization, storage, or retention [6]. From a pasture-monitoring perspective, many of these disturbances originate from shifts in parameters that RS and PS technologies can estimate with varying accuracy. This means that deviations in these pasture attributes can serve as an early indirect indicator of metabolic instability, allowing risk to be detected before clinical signs appear.

Additional contributing factors include nutrient imbalances or antagonistic interactions, elevated nutrient losses, increased metabolic demands driven by physiological or environmental stressors, dysregulation of metabolic control mechanisms, dehydration, and exposure to dietary or environmental toxins. Other deleterious factors in pasture plants encompass a wide range of chemical and physical components that limit the ability of grazing animals to achieve their full productive potential. These include compounds that predispose animals to ruminal bloat, excessive nitrate accumulation that can impair oxygen transport, and naturally occurring plant toxins such as glycosides, alkaloids, and neurotoxins that disrupt normal metabolic and neurological functions. In addition, mycotoxins produced by fungi or endophytic organisms may induce conditions such as facial eczema, photosensitization, and other endophyte-associated disorders. Pasture plants may also contain anti-mineral and anti-vitamin factors that reduce nutrient bioavailability, as well as structural features including high lignin content, tannins, thorns, awns, and burs, which can restrict voluntary feed intake and hinder digestive efficiency [36].

Consequently, metabolic disorders encompass—but are not limited to—nutritional and acquired conditions, even though these are typically the primary focus in animal production practice. While specific nutrient deficiencies or excesses can experimentally induce well-defined syndromes, such as iodine deficiency leading to goiter or excessive calcium intake causing metastatic calcification, field conditions on farms more commonly involve complex interactions among multiple nutrient imbalances [6]. Among the nutrient-related disorders directly linked to pasture conditions, hypomagnesemia (grass tetany) represents a prominent example where forage quality, soil chemistry, and animal physiology intersect. Grazing cattle are particularly susceptible during periods of rapid pasture growth, when lush grasses contain low magnesium (Mg) and elevated potassium and nitrogen concentrations that depress Mg absorption in the rumen. This imbalance has been identified as a major cause of morbidity and mortality in beef herds grazing improved natural grasslands [40]. Because forage Mg content reflects both plant composition and underlying soil characteristics, the risk of hypomagnesemia is further amplified on acidic soils where low pH limits grass productivity and reduces plant-available Mg. Improving soil conditions through liming—especially with Mg-rich lime—raises soil pH and increases soil Mg concentrations, thereby enhancing the Mg content of forage and providing an effective preventive strategy against hypomagnesemia in grazing cattle [41]. Importantly, several of these risk factors correlate with pasture conditions that can be monitored remotely. For example, lush, high-CP, low-fiber swards, common in early spring or legume-rich paddocks, are associated with increased risk of frothy bloat. Conversely, mature, lignified pastures with high NDF and low energy availability predispose animals to negative energy balance and subclinical ketosis. Because these nutritional patterns are linked to phenological stage, canopy structure and spectral signatures, sensing technologies can help identify high-risk conditions without destructive sampling.

With this in mind, the prevention of alimentary metabolic disorders is fundamental to optimizing productivity and economic returns in breeding livestock. In high-producing dairy cows for example, the risk of such disorders can be substantially reduced through the provision of well-balanced diets and the use of high-quality forages that are specifically formulated to meet the animals’ physiological and production requirements [42], which bring us again to pasture monitoring technologies as tools for nutritional and metabolic disorder prevention in grazing ruminants.

## 7. AI, IoT and ML Role in Predictive Models

The value of AI in precision grazing depends fundamentally on the quality, diversity, and temporal continuity of the input data. Modern pasture monitoring systems therefore integrate heterogeneous information collected from satellite imagery, UAV observations, proximal sensing, weather stations, soil sensors, and, increasingly, animal-borne monitoring devices. These complementary datasets constitute the foundation upon which predictive models and DSSs are developed.

The incorporation of IoT technologies into agricultural systems is transforming food production, management, and distribution processes. Through the integration of intelligent sensor networks, robust communication frameworks, distributed computing infrastructures, and AI, IoT-enabled smart farming platforms support precise, data-driven monitoring and optimized management of agricultural inputs and resources [43].

ML algorithms adaptively learn from experimental datasets, enabling the identification of complex relationships and the derivation of actionable insights [44] extracting meaningful patterns from data, which can be achieved using either supervised or unsupervised learning strategies. Supervised learning techniques—including support vector machines, decision trees, random forests, Naive Bayes regressors, and multilayer perceptron neural networks—rely on labelled datasets to develop predictive models tailored to specific tasks [1]. For example, Zwick et al. [45] demonstrated that combining ML with high-resolution multispectral remote sensing data allows accurate prediction of pasture biomass and key nutrient quality indicators such as CP and digestibility in tropical grasslands; their work highlights the strong performance of ensemble learners like random forests and meta-learning stacks for supporting data-driven, real-time grassland monitoring and sustainable management decisions.

**Figure 6:**
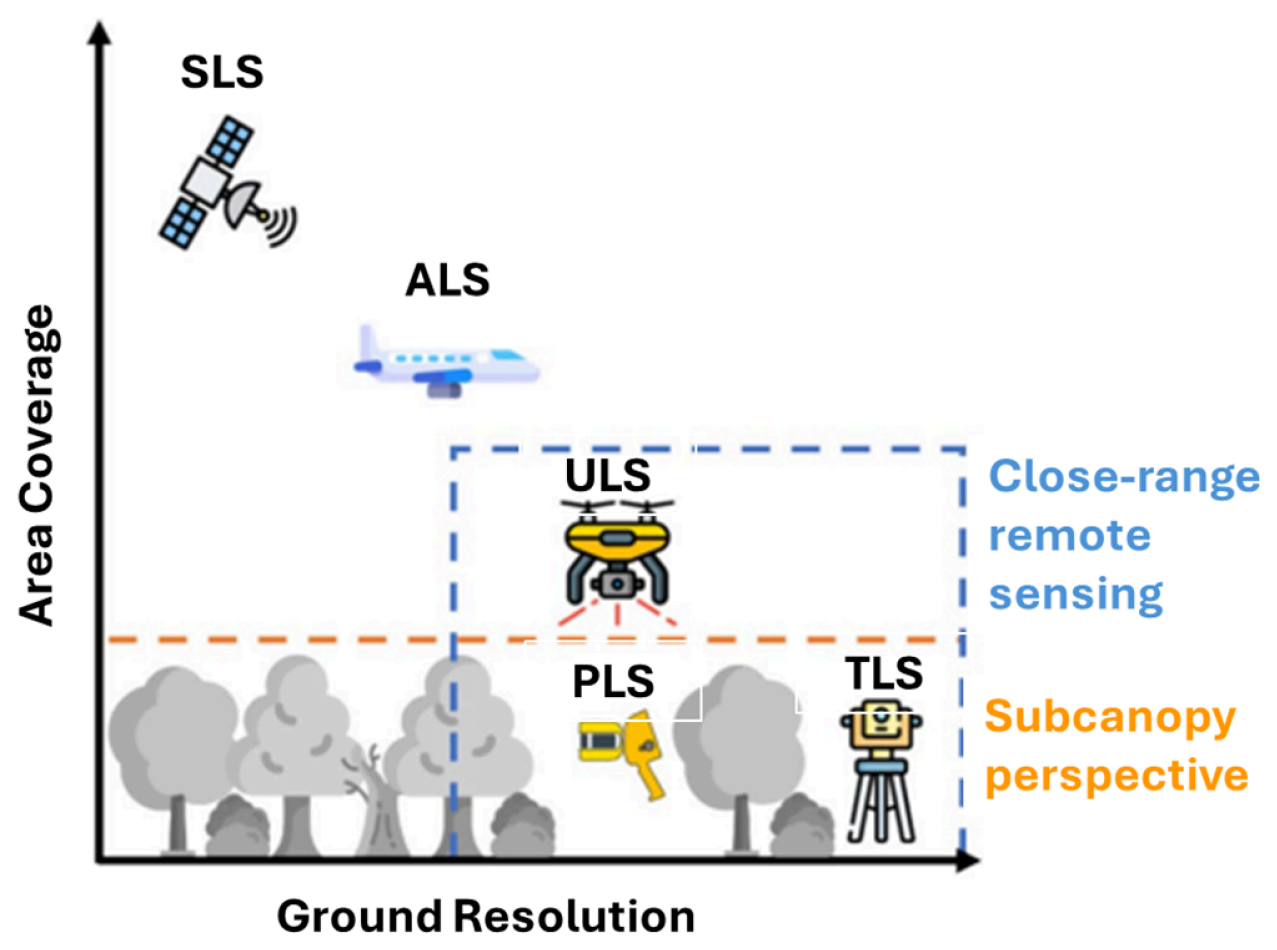
Overview of LiDAR platforms and their respective perspectives in a forest structure for biodiversity assessments. ALS—aerial laser scanning; PLS—personal laser scanners; SLS—satellite laser scanning; TLS—terrestrial laser scanners; ULS—uncrewed aerial laser scanning [48].

The integration of these three key components (ML, AI and IoT) enables real-time data collection, analysis, and decision-making [46], allowing predictive models development. IoT sensors constitute the backbone of precision agriculture, creating a continuous pipeline that transmits real-time field data to cloud-based platforms for analysis and decision-making [47] through hardware that includes soil sensors (moisture, pH, and electrical conductivity), weather monitoring stations (temperature, humidity, solar radiation, and wind speed) and UAVs (equipped with multispectral and hyperspectral cameras) [43]. Integration with RS technologies (such as satellites, aerial imagery, and LiDAR platforms—Figure 6; [48]) complements this data collection system by providing broader spatial coverage and temporal frequency, allowing large-scale assessment of crop health, growth patterns, and environmental variability beyond the limits of in-field sensors and UAV flights.

**Figure 7:**
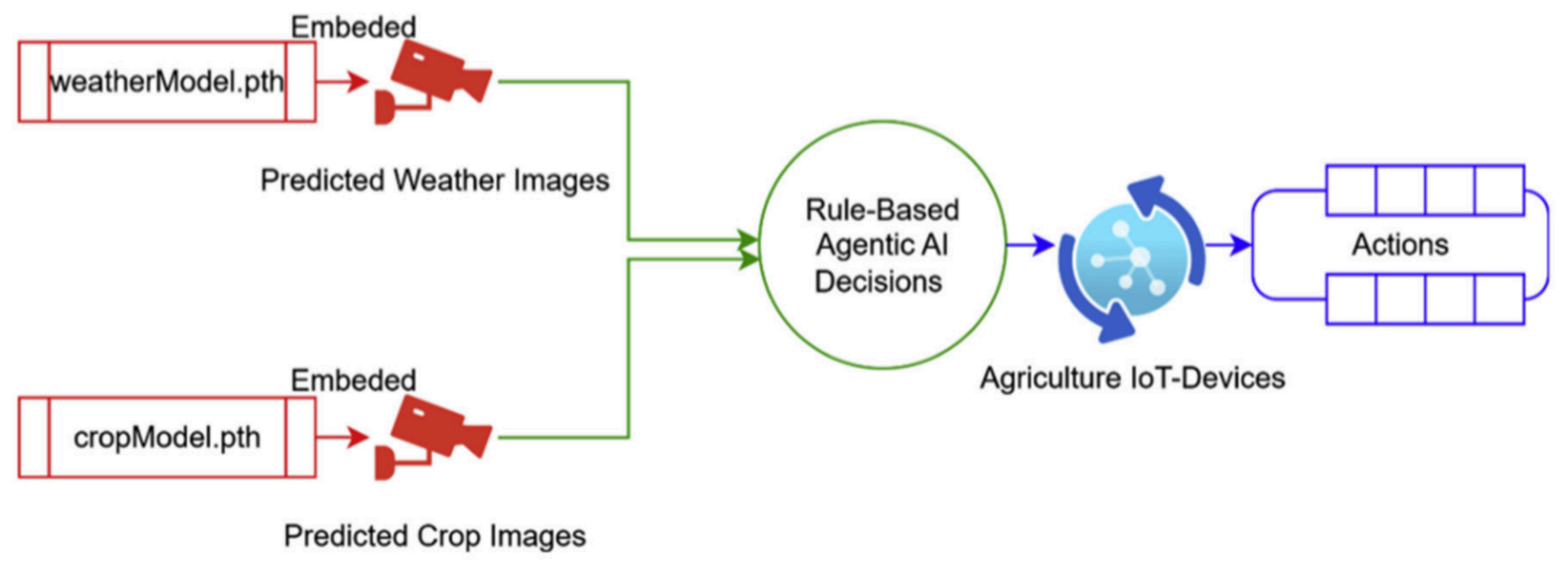
IoT-enabled smart agriculture simulation with agentic-AI decision support and deep learning integration [49].

By integrating multiple types of sensors, comprehensive monitoring systems can be established to meet a wide range of agricultural needs. Ensuring efficient data transmission is critical, particularly in areas with limited connectivity, to maintain the reliability of these networks. The incorporation of renewable energy sources further enhances both the sustainability and operational stability of IoT deployments. At the same time, advanced neural network models improve the accuracy of weather forecasts, which are essential for effective agricultural planning. Although these models require substantial computational resources, modern cloud infrastructure enables their practical implementation, allowing real-time analysis and decision-making [47]. In recent work, Tariq et al. [49] proposed an edge-centric framework that enables low-cost, robust, and autonomous decision-making. Their approach demonstrates that actionable context can be achieved by fusing two camera-derived signals—crop identity and weather classification—without introducing additional specialized sensors. Lightweight MiT-B0 vision models operating on low-resolution inputs were shown to run efficiently on resource-constrained edge devices, significantly reducing latency, bandwidth usage, and cloud dependency. System robustness and maintainability were further enhanced using two decoupled vision models, eliminating the need for multisensor calibration and allowing independent updates, optimization, or replacement (Figure 7).

The framework extends beyond perception by integrating a rule-based agentic AI layer that translates model predictions into real-world actions. This decision engine supports both joint-condition rules and fallback single-condition logic, enabling coordinated actuation across multiple IoT components, including smart irrigation systems, NDVI sensors, frost alarms, drones, and pest detection units [49].

In a different approach, Chourlias et al. [50] demonstrated that ML-based virtual sensors can reliably estimate key environmental variables, such as soil moisture, temperature, humidity, light, and UV radiation, providing a scalable and cost-effective alternative to physical sensors in smart farming. Among the evaluated models, the Light Gradient Boosting Machine achieved the highest accuracy, with prediction errors below 1% in most cases, demonstrating the reliability of virtual sensing. Additionally, the results indicate that, under certain conditions, virtual sensors can be powered solely by open weather data, highlighting the potential for hardware-independent monitoring solutions.

In a recent systematic review, Miller et al. [51] explain the emerging trends in smart agriculture and emphasizes the increasing role of edge AI, blockchain, and robotic systems in enhancing the efficiency, scalability, and autonomy of agricultural operations. Edge AI enables local data processing and real-time decision-making directly at the sensor or device level, reducing latency and dependence on continuous cloud connectivity. Blockchain technologies are highlighted to ensure secure, transparent, and decentralized management of agricultural data, while robotics supports automated field operations such as precision planting, monitoring, and harvesting. Despite these advances, the review reveals a critical research gap in the calibration and validation of existing and newly developed sensors, which remains essential for ensuring data reliability and consistency. Addressing this gap is fundamental to enabling the development of novel sensing hardware and robust data collection systems capable of delivering high-quality real-time data [51].

## 8. Decision-Support Frameworks and Management Support Systems

DSSs are increasingly being adopted in PA, particularly for forage and grassland management. A wide range of applications have demonstrated significant benefits across areas such as nutrient management, insect pest control, and livestock production [29]. For pasture management, DSSs have become essential tools for farms that rely heavily on pasture as a primary feed source. These systems improve pastureland management by optimizing pasture supply, quality, and utilization, which in turn enhances farm profitability. Common DSS tools include the pasture wedge, pasture budget, and spring and autumn rotation planners, which assist farmers in short- to medium-term management decisions. More advanced pasture DSSs can incorporate historical pasture growth data to estimate long-term production capacity, supporting strategic choices such as optimal stocking rates and paddock reseeding schedules—factors closely linked to the economic performance of pasture-based operations [52].

Many DSSs for pasture monitoring are now available as web-based applications or mobile platforms, enabling farmers to monitor and manage operations remotely. As examples, Ali and Kaul [29] referred to several DSSs in their recent review article, including AgroMANAGER^®^, which supports farm record-keeping and online monitoring, and open-source tools in Europe such as MesParcelles^®^ in France, AgrarCommander^®^ in Austria, NMP Online^®^ in Ireland, MarkOnline^®^ in Denmark, and Web Module Düngung^®^ in Germany. Additionally, the European project GrassQ^®^ applies this approach to precision grassland management, integrating proximal sensor measurements and satellite data to provide actionable insights directly through accessible digital platforms [29]. Despite this, and as mentioned above, decision support frameworks and management support systems can be built to incorporate more variables. Although the implementation of DSSs varies among production systems, most platforms follow a common decision workflow. Initially, information from RS, PS, IoT devices, and animal-based sensors is acquired and integrated with environmental, agronomic, and animal production data. These inputs are subsequently processed using empirical models, mechanistic approaches, or ML algorithms to estimate pasture availability and quality, predict animal nutritional requirements, and identify potential management constraints. Based on these outputs, the DSS generates practical recommendations such as grazing allocation, stocking-rate adjustment, pasture rotation, supplementation planning, or irrigation scheduling. The effectiveness of these systems ultimately depends on the quality of sensor-derived information, the robustness of predictive models, and the translation of these outputs into actionable management decisions. Table 4 summarizes the main types of DSSs described in the literature according to their required inputs, decision outputs, target users, management actions, and current limitations.

In their study, Asher and Brosh [5] discussed a Herd-Management System which integrates RS technology with behavioral and location data from GPS-equipped collars on grazing cows. The system continuously collects data on individual cows’ activity (grazing time, resting, walking), herd movement patterns, and spatial position, and analyzes these data to support grazing and herd management decisions [5]. Niloofar et al. [53] proposes a more holistic framework for assessing DSSs in livestock farming, focusing on environmental, economic, and social sustainability dimensions [53]. Panda et al. [54] developed a mobile-phone-based automated DSS aimed at improving livestock management for resource-poor farmers, particularly in southern Africa. They focused on optimizing the production of anti-parasitic, tannin-rich forage legumes adapted to local environmental conditions, monitoring animal health through RFID-supported telemetry to detect early signs of disease, and integrated this information into a smartphone application to provide real-time alerts. The DSS also incorporates educational components to train farmers on sustainable worm management and best practices, with the overall goal of enhancing livestock productivity and supporting the economic well-being of farming communities [42].

Reeves et al. [55] developed a system that, by leveraging increased data availability and computational power, enabled the calculation of stocking rate estimates over large areas while simultaneously accounting for both spatial and temporal variations in forage production, capabilities that were not previously available at this scale. This research gave life to the StockSmart^®^ calculator [55].

**Figure 8:**
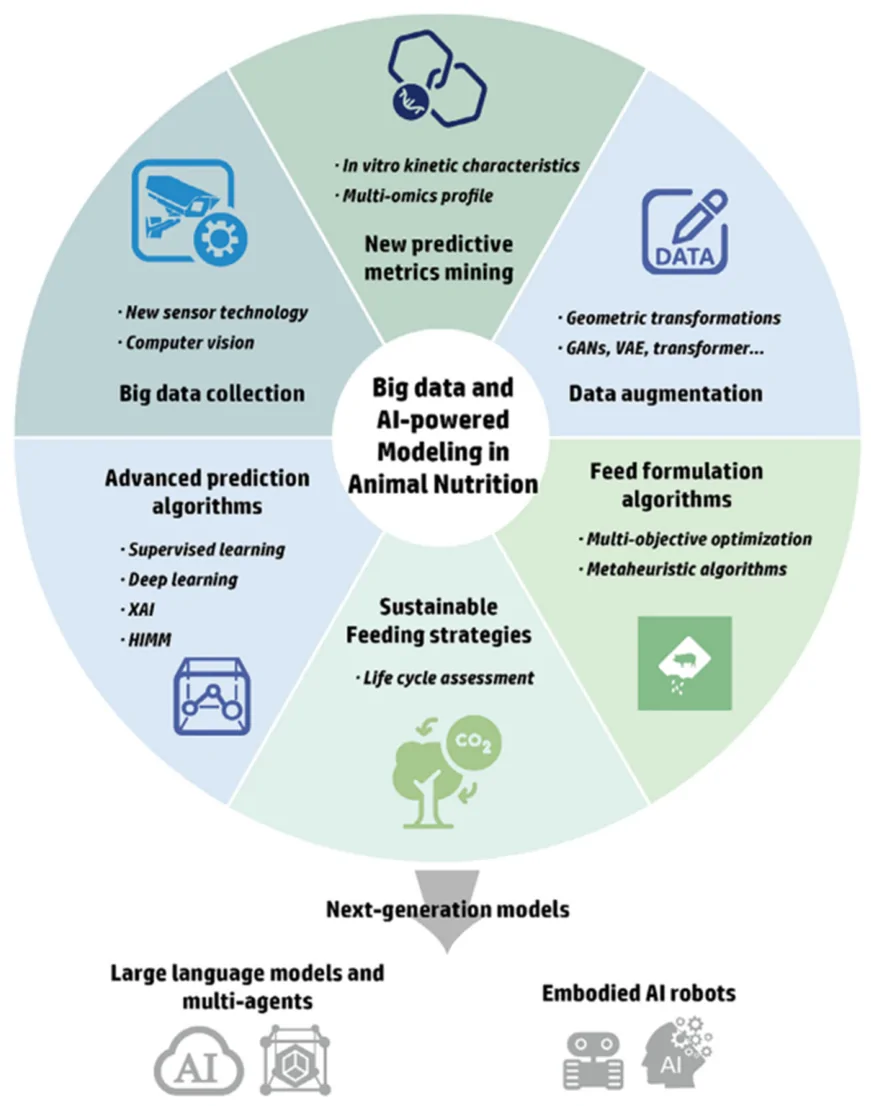
Schematic of big data and artificial intelligence (AI)-powered modeling in animal nutrition and feeding. GANs: generative adversarial networks; HIMM: hybrid intelligent mechanistic models; VAE: variational autoencoders; XAI: explainable artificial intelligence [56].

For Zhang et al. [56] new sensor and computer vision technologies laid a solid foundation for continuous, high-resolution big data collection in animal nutrition and feeding, overcoming the limitations of traditional manual measurements. Wearable, environmental, ingestible, in-line, and bolus sensors enable real-time monitoring of physiological, behavioral, intake, and environmental parameters, while RFID supports individual animal identification and data integration. In parallel, NIR, Mid-Infrared Spectroscopy (MIR), and hyperspectral imaging allow rapid, on-site assessment of feed nutrient composition, and computer vision provides non-invasive estimation of feed intake, body weight, body condition, and animal behavior. As indicated in Figure 8, together these approaches can generate detailed and continuous data streams that provide stronger evidence of how animals respond to feed and nutrient supply, despite remaining challenges related to cost, data processing, and system integration [56].

Recent Mediterranean-focused reviews further reinforce the strategic role of RS within broader pasture management and decision-support contexts. Patera et al. [57] highlights how multispectral, hyperspectral, radar, and LiDAR data—combined with ML approaches such as random forest, support vector machines, and gradient boosting algorithms—are increasingly used to support large-scale monitoring of pasture biomass, vegetation dynamics, and land-use changes. Their synthesis emphasizes that RS outputs can inform adaptive grazing strategies, degradation assessment, and climate-resilient management planning, particularly in heterogeneous Mediterranean rangelands. However, while these frameworks significantly advance landscape-scale monitoring and sustainable pasture management, their integration with animal-level nutritional indicators and metabolic risk assessment remains limited. This gap underscores the importance of coupling remote sensing-based pasture evaluation with animal-centered DSS architectures capable of translating vegetation dynamics into actionable supplementation and health management decisions.

**Figure 9:**
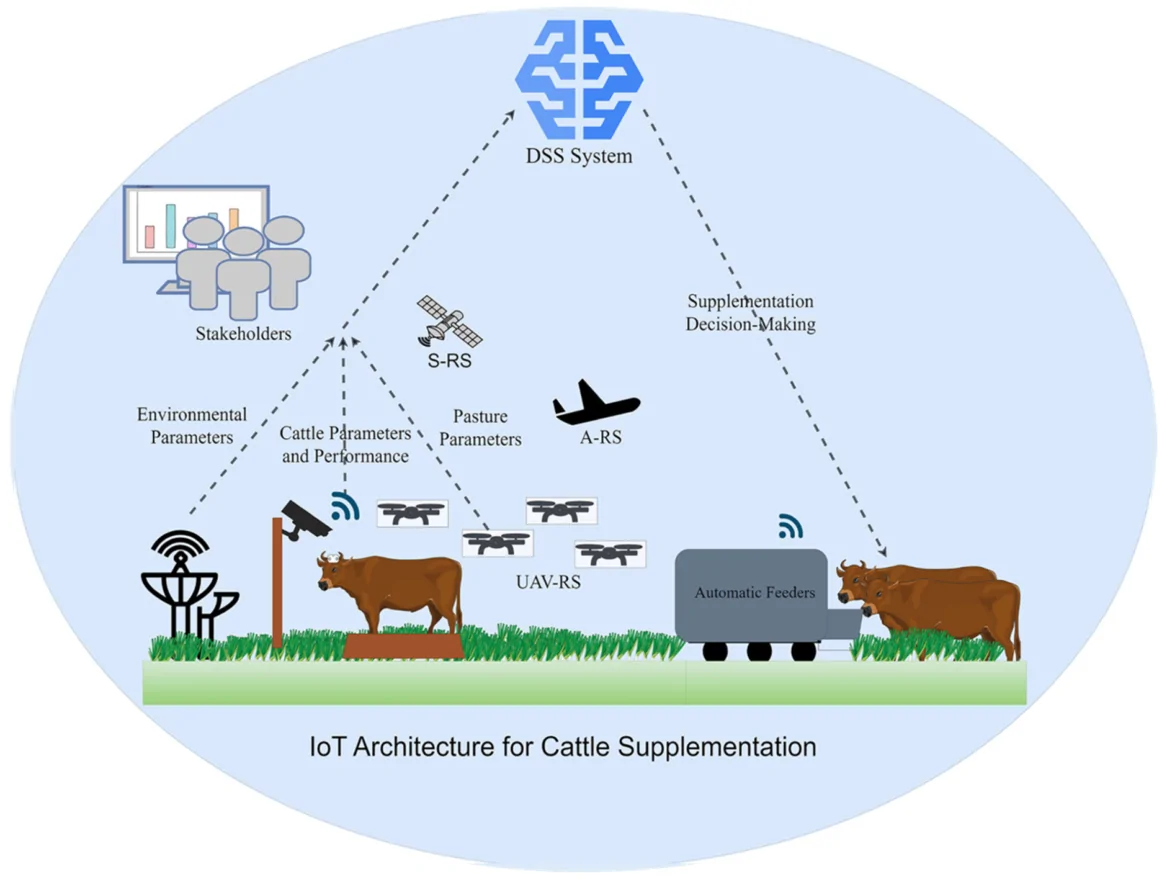
A high-level view of IoT architecture for cattle supplementation basis for the decision support system for cattle supplementation [39].

Defalque et al. [39] identify RS, PS, livestock welfare wearables, Walk-Over Weighting (WOW) scales, and climate stations as state-of-the-art technologies for estimating supplementation related parameters. An IoT architecture that integrates these heterogeneous data sources constitutes a key tool for the implementation of a DSS for cattle supplementation. Sensor-derived data are systematically organized within the control and storage layer, which comprises a cloud-based database and dedicated services for data storage, management, and retrieval. These integrated datasets are subsequently employed to train ML models for the estimation of pasture quality parameters such as CP, ADF, and total digestible nutrients (TDN) and to support the decision-making process aimed at defining optimal supplementation strategies. Moreover, the decision-making process can be coupled with actuation mechanisms, enabling the automatic configuration of supplementation levels delivered by a Programmable Automatic Feeder (PAF) according to predefined performance targets. Ultimately, this framework allows the dynamic adjustment of supplementation requirements necessary to achieve predefined targets of average daily gain (ADG) in cattle. Figure 9 details the DSS described in this study, illustrating the integrated IoT architecture for cattle supplementation. The figure highlights the flow of data from environmental, pasture, and animal related sensing technologies to the DSS, as well as the resulting supplementation decision-making process and its interaction with automatic feeding systems.

Despite all these advances, delineating sward heterogeneity in mixed grasslands remains challenging due to spectral similarities among species and the limitations of remote indices such as NDVI [29]. To overcome these constraints, Ali and Kaul [34] recommend the use of diversity interactions models combined with spectral unmixing approaches and hyperspectral imaging techniques, including super pixel segmentation and multilevel data fusion procedures. They emphasize that sustainable grassland management requires a multifunctional framework capable of balancing ecological, socioeconomic, and policy trade-offs, including implementation costs and ecosystem service benefits [54]. The integration of digital technologies for data acquisition and augmentation, such as electronic identification systems, RFID, soil and plant sensors, smartphones, high-throughput proximal and remote sensing platforms, ML, AI-based algorithms, and automatic application controllers, is considered essential for improving grassland monitoring and management across diverse environments [29] improving sustainable farming and reducing metabolic and nutritional disorders in grazing ruminants.

Table 5 consolidates the main sensing technologies used in pasture monitoring and aligns them with the forage attributes each system can estimate, together with the physiological mechanisms through which these attributes influence rumen function and metabolic balance. By structuring the information across proximal sensors, remote platforms, multisensor fusion, and analytical approaches, the table clarifies how different technologies capture complementary aspects of pasture structure, composition, and nutritional value.

Proximal sensors (RPM, Grassmaster^®^, OptRx) provide high-resolution measurements of structural and biochemical attributes that determine digestibility and intake dynamics. Remote sensing platforms (UAV and satellite) extend these assessments to larger spatial scales, capturing canopy structure, biochemical composition, and moisture status with increasing predictive reliability. Multisensor fusion approaches improve trait estimation by combining structural and spectral information, particularly for CP, NDF, and WSC. Finally, ML-based and DSM approaches integrate multitemporal and multisensor data to model pasture quality and metabolic risk more holistically.

From this synthesis, it becomes evident that integrating measurements across scales and sensor types enables earlier detection of shifts in key traits—such as CP, NDF, ADF, WSC, and ME—that precede metabolic instability in grazing ruminants. Because these traits are mechanistically linked to disorders such as hypomagnesemia, SARA, ketosis, and energy deficiency, their continuous monitoring provides actionable indicators for timely management adjustments.

In practical terms, combining proximal, UAV, satellite, and model-based approaches support a more informed and anticipatory grazing strategy, allowing managers to adjust stocking rate, supplementation, and pasture allocation before nutritional imbalances escalate into metabolic disorders. This integrated monitoring framework therefore strengthens preventive management and enhances metabolic resilience in grazing systems.

Scalability and transferability remain critical considerations for the practical implementation of sensing-based pasture management. Scalability should be considered not only in terms of spatial coverage, but also in relation to economic accessibility, infrastructure requirements, connectivity, technical expertise, calibration needs, and data-processing capacity. In resource-constrained or smallholder systems, mobile- and web-based DSSs combined with relatively simple sensing approaches may provide more accessible solutions, as illustrated by systems developed for resource-poor farmers using smartphones, RFID-supported telemetry, and automated alerts. In medium-scale farms, satellite imagery, PS, and farm-level DSSs can provide a balance between spatial coverage and operational complexity. Large commercial operations may be able to integrate UAVs, extensive IoT networks, animal-based sensors, and multisource data-fusion systems to support high-resolution and continuous monitoring. However, technological scalability does not necessarily imply economic or operational scalability. In particular, site-specific calibration, connectivity, data-processing requirements, and the transferability of predictive models across pasture types and environmental conditions may constrain adoption across production systems. Consequently, scalable solutions should be designed around modular architectures that allow sensing intensity and analytical complexity to be adapted to farm size, infrastructure, and management capacity.

## 9. Limitations of This Review

Although this review followed a structured and transparent literature search strategy, it was conducted as a narrative review rather than a formal systematic review. Consequently, the study selection process may be subject to selection bias, as articles were included based on their scientific relevance and contribution to the objectives of the review rather than through predefined systematic-review protocols. In addition, only peer-reviewed publications written in English were considered, which may have resulted in the exclusion of relevant studies published in other languages.

The literature search focused primarily on studies published between 2021 and 2025 to capture the most recent advances in RS, PS, UAV, and AI applied to pasture monitoring. Although this approach ensured that the review reflects the current state of the art, it may have limited the inclusion of older foundational studies and region-specific research that remain relevant to the field.

Furthermore, this review did not include a quantitative meta-analysis or formal statistical comparison of study outcomes. Differences in experimental designs, sensing platforms, pasture types, environmental conditions, calibration procedures, and performance metrics reported across the literature limited direct quantitative comparisons among studies. Consequently, the conclusions presented should be interpreted as a qualitative synthesis of the current evidence rather than as quantitative estimates of technology performance.

Economic feasibility represents an additional limitation of the current evidence. Sensor-based pasture monitoring involves costs associated with acquisition, installation, calibration, maintenance, connectivity, data processing, software, and user training, which vary substantially with sensing technology, farm size, infrastructure, and degree of automation. Although potential benefits include improved pasture allocation, stocking-rate decisions, supplementation, labor efficiency, and earlier identification of nutritional risk, the reviewed literature does not provide sufficiently consistent farm-level economic data to establish general cost–benefit ratios or return-on-investment estimates. In particular, direct evidence that sensor-based monitoring reduces the economic impact of metabolic disorders remains limited.

Another important limitation concerns the transferability and scalability of sensing technologies across production systems. The reviewed studies differ considerably in farm size, pasture composition, climatic conditions, management intensity, infrastructure availability, and sensor configuration. Consequently, a technology or predictive model demonstrating satisfactory performance in one production context cannot necessarily be assumed to maintain the same performance or economic feasibility in another. This is particularly relevant for biochemical pasture traits, for which calibration and model transfer remain important challenges.

Despite these limitations, the review provides an up-to-date and comprehensive synthesis of recent developments in pasture quality monitoring, identifies current knowledge gaps, and highlights future research directions for integrating sensing technologies into DSSs for sustainable grazing management.

## 10. Conclusions

This review synthesizes recent advances in pasture monitoring, precision grassland management, RS, and DSS applications for grazing livestock systems. Recent evidence shows that sensing modalities used vary in their sensitivity to structural, biochemical, and moisture-related traits, and that combining these traits improves the capacity to anticipate nutritional imbalances. The literature also consistently shows that integrating RS with PS for automated data acquisition improves the understanding of spatial-temporal variability in pasture growth, quality, and utilization, enabling the building of web-based or mobile DSSs. These tools support more informed short, medium, and long-term management decisions related to grazing management, stocking rate adjustment, and supplementation strategies, with clear implications for farm productivity and sustainability. The increasing availability of high-resolution data, combined with advances in data processing and modeling capacity, enables decision-making at spatial and temporal scales that were previously unattainable in pasture-based systems. This is particularly relevant because shifts in CP, NDF, WSC, ME and fiber fractions often precede metabolic instability, and their early detection enhances the preventive value of DSSs.

The review also highlights the growing convergence between pasture monitoring technologies and animal-based sensing. Wearable sensors, RFID-based identification, and environmental monitoring systems strengthen the link between pasture conditions, animal behavior, and physiological responses. This integration enhances the capacity of DSSs to support animal health and nutritional management by moving beyond static indicators toward dynamic, real-time assessments. In this context, DSSs increasingly function as integrative platforms that combine pasture, animal, and environmental data rather than isolated decision tools. This multilayer perspective aligns with current research showing that metabolic disorders emerge from interacting nutritional drivers rather than single isolated parameters.

A key conclusion of this review is that novel sensing technologies, particularly when calibrated and integrated with RS data, represent a promising approach to improving pasture characterization and nutritional management in grazing ruminants. Improved estimation of biomass availability and forage quality can support more informed grazing and supplementation decisions, thereby helping to mitigate nutritional imbalances. However, direct evidence linking sensor-based pasture monitoring to reduced metabolic disorder incidence remains limited. Establishing this relationship will require validation of pasture sensing outputs against animal-level responses, including feed intake, rumen function, productive performance, mineral status, blood metabolites, and health records. The literature indicates that predictive accuracy is generally higher for structural traits than for biochemical parameters such as CP, NDF, and WSC, reinforcing the need for multimodal sensing strategies and integrated DSSs. Ultimately, the transition from pasture monitoring to health-oriented grazing management will depend on the successful integration of sensing technologies with animal-level monitoring and evidence-based decision support systems capable of translating data into practical management actions.

## 11. Future Research

Future research should prioritize the development, calibration, and validation of emerging sensor technologies in combination with RS for pasture and animal monitoring under diverse grazing conditions. Given that different sensing modalities vary in their sensitivity to structural versus biochemical traits, calibration must explicitly account for these differences to avoid systematic bias in DSS inputs. Particular attention is needed for sensors capable of capturing pasture quality parameters relevant to ruminant nutrition—including energy, protein, fiber fractions, and secondary compounds—and for their calibration against satellite- and UAV-derived indicators. Calibrating these sensors across heterogeneous grasslands and mixed swards remains essential to ensure accurate inputs for DSS-based supplementation and grazing management.

Further work is required to strengthen the integration of pasture monitoring with animal health and nutrition indicators within DSSs. Linking pasture dynamics derived from RS with real-time animal responses—such as behavior, intake proxies, and physiological status—offers significant potential to anticipate nutritional stress and metabolic disorders before clinical symptoms occur. This requires linking pasture-derived indicators such as CP, NDF, ME and WSC with animal-level proxies of intake, rumen function and energy balance, an area where current evidence remains fragmented. Such integration supports the transition from reactive to preventive management strategies in grazing systems.

Research should also address the scalability and accessibility of DSSs across farms with different sizes, infrastructure levels, and production objectives. Mobile- and web-based platforms may facilitate adoption in resource-constrained systems, whereas more complex multisensor architectures may be appropriate where infrastructure and technical capacity allow. Cost-effective sensor deployment, standardized data protocols, interoperable architectures, and transferable calibration procedures are necessary to enable adoption across farms of different sizes and production contexts. In parallel, user-centered design and training components remain critical to ensure that DSS outputs translate into actionable management decisions. Future systems should be capable of integrating heterogeneous data streams—proximal, UAV, satellite, and animal-based—into unified nutritional indicators that are interpretable by farmers.

Future studies should also quantify the economic feasibility of integrated sensing systems under different farm sizes and production contexts. Cost–benefit analyses should consider not only equipment acquisition and maintenance costs, but also calibration, connectivity, data processing, software, labor requirements, and training. These analyses should be combined with measurable outcomes such as changes in supplementation costs, labor requirements, stocking-rate efficiency, pasture utilization, animal productivity, and the incidence or severity of nutritional and metabolic disorders. Such farm-level evaluations would provide the evidence required to determine when the economic benefits of sensing technologies outweigh their implementation costs.

Finally, future studies should explore the application of advanced analytics, including ML and AI-based models, to improve data fusion, uncertainty handling, and predictive capacity within DSSs. Advanced analytics are particularly needed to reconcile discrepancies between structural and biochemical predictions, and to model how combinations of pasture traits contribute to metabolic risk. When combined with calibrated sensors and robust pasture monitoring, these approaches can enhance supplementation strategies, improve animal health outcomes, and contribute to more resilient and sustainable grazing ruminant systems.

## Figures and Tables

**Table 1:** Summary of grass quality indicators applied in recent studies.

Quality Indicator (Units)	What is Measured	Application for Forage Monitoring	Ref.
Moisture content/ Water content (%)	Proportion of water in plant material	Determines optimal harvest timing and feed conservation quality	[14]
DM (kg ha^−1^)	Total solids in forage	Standardizes forage quality assessment across fresh and processed samples	[15]
OM (%)	Combustible fraction of dry matter	Indicator of nutritive fraction excluding ash	[16]
CP (%)	Total nitrogen × 6.25	Core indicator of forage nutritive value	[17]
N (%)	Nitrogen concentration in plant tissue	Basis for crude protein estimation and plant nutritional status	[17]
NDF (%)	Total cell wall fraction	Structural indicator related to forage bulk and maturity	[17]
ADF (%)	Cellulose + lignin fraction	Indicator of potential digestibility and forage maturity	[17]
ADL (%)	Lignin concentration	Proxy for indigestible plant material and advanced maturity	[17]
Fat/Lipid content (%)	Ether-extractable lipids	Minor forage quality component, difficult to predict with NIR	[18]
WSC (%)	Soluble sugars in plant tissue	Indicator of readily available energy and forage conservation quality	[19]
Ash content (%)	Total mineral residue after combustion	Indicator of mineral content and possible soil contamination	[18]
PQI	Composite index integrating multiple quality traits	Integrative grass quality monitoring metric for spatial and temporal assessment	[9]
Spectral vegetation indices (NIR-derived)	Reflectance-based indices linked to chemical traits	Rapid, non-destructive monitoring of forage quality proxies	[19]

Ref.—reference; DM—dry matter; OM—organic matter; CP—crude protein; N—nitrogen; NDF—neutral detergent fiber; ADF—acid detergent fiber; ADL—acid detergent lignin; WSC—water-soluble carbohydrates; PQI—Pasture Quality Index; NIR—near-infrared.

**Table 2:** Comparison of remote and proximal sensing technologies for pasture monitoring.

Pasture Indicator	Detectable by RS	Detectable by PS	Validation Required	Operational Readiness
Biomass	✓✓✓	✓✓	Low	High
Height	✓✓✓	✓✓✓	Low	High
CP	✓	✓✓✓	High	Moderate
NDF	✓	✓✓✓	High	Moderate
ADF	✓	✓✓	High	Moderate
WSC	✓	✓✓	High	Low
Moisture	✓✓✓	✓✓	Moderate	High
Minerals	✗	✓	Very high	Low

RS—remote sensing; PS—proximal sensing; CP—crude protein; NDF—neutral detergent fiber; ADF—acid detergent fiber; WSC—water-soluble carbohydrates; ✓ to ✓✓✓—three-levels on a semi-quantitative scale of performance or applicability; ✗—not detectable.

**Table 3:** Comparative assessment of the principal sensing technologies used for pasture quality monitoring according to their capability for estimating biomass, CP, and NDF, together with their relative cost-effectiveness.

Technology	Biomass	CP	NDF	Cost-Effectiveness	Calibration Requirement	Monitoring Frequency
Sentinel-2	★★★★★	★★	★	★★★★★	★★	★★★★★
Landsat	★★★★	★	★	★★★★★	★★	★★★★
UAV RGB	★★★★	★	★	★★★	★★	★★★
UAV Multispectral	★★★★★	★★★	★★	★★★	★★★	★★★
UAV Hyperspectral	★★★★★	★★★★★	★★★★★	★	★★★★★	★★
LiDAR	★★★★★	★	★	★★	★★	★★★
RPM	★★★★	★★★	★★	★★★★★	★★	★★★★★

CP—crude protein; NDF—neutral detergent fiber; UAV—unmanned aerial vehicles; RGB—red, green and blue; LiDAR—light detection and ranging; RPM—rising plate meter; ★ to ★★★★★—five-levels on a semi-quantitative scale of performance.

**Table 4:** Main types of DSSs according to their required inputs, decision outputs, target users, management actions, and current limitations.

DSS Type	Required Input Data	Decision Output	Management Action	Target User	Main Limitation	Base Reference
Grazing allocation	Biomass, grazing rotation	Grazing order	Paddock allocation	Farmers	Biomass only	[52]
Pasture budgeting	Biomass, stocking rate, growth rate	Feed balance	Supplement planning	Farmers	Requires frequent updates	[52]
Seasonal grazing planning	Biomass, growth rate	Rotation schedule	Early grazing management	Dairy farmers	Seasonal applicability	[52]
Farm management	Pasture biomass, satellite imagery, PS	Monitoring and management recommendations	Precision pasture management	Farmers	Platform-specific implementation	[29,53]
Herd management	GPS collars, behavioral data, RS	Grazing and herd management	Grazing optimization	Farm managers	Requires animal instrumentation	[5]
Animal health	RFID, Telemetry, Forage data	Animal health alerts	Parasite and forage management	Smallholder farmers	Infrastructure dependence	[54]
Stocking rate	Biomass, pasture growth, climate	Stocking rate estimation	Grazing allocation	Farm managers	Requires historical datasets	[55]
Animal nutrition	Biomass, CP, NDF, animal requirements, intake and sensor-derived animal data	Feed allocation and supplementation strategy	Nutritional management	Nutritionists/Farmers	Requires accurate pasture and animal data integration	[56,57]

DSS—decision support system; RFID—radio frequency identification; PS—proximal sensing; RS—remote sensing; GPS—global positioning system; CP—crude protein; NDF—neutral detergent fiber.

**Table 5:** Integrated overview of proximal, remote, and fused sensing technologies, pasture quality indicators, mechanisms of action, predictive performance, and associated metabolic risks in grazing ruminants.

Sensor/Technique	Pasture Parameters	Mechanism of Action	Predictive Reliability	Ref	Associated Metabolic Disorders
Proximal Sensors					
Rising Plate Meter (RPM)	SSH, biomass, DM	DM → determines energy availability and intake potential	R^2^ ≈ 0.70–0.85; RMSE ≈ 0.25 kg DM m^−2^	[58]	Under- or over-grazing → acidosis; ketosis
Electronic Capacitance Probe (Grassmaster^®^)	GM, DM, NDF	NDF → determines rate of digestion and intake potential	R^2^ = 0.92 (GM); R^2^ = 0.91 (DM); RMSE ≈ 647 kg ha^−1^	[57]	Low DM → energy deficiency; low NDF → ruminal acidosis
Optical Proximal Sensor (OptRx/GreenSeeker)	CP, PMC, NDF	CP → microbial protein synthesis; NDF → intake potential	PMC (R^2^ = 0.88); CP (R^2^ = 0.67); NDF (R^2^ = 0.50)	[27]	Low CP → hypomagnesemia; high NDF → SARA
Remote Sensors					
UAV Hyperspectral/LiDAR	CP, NDF, ADF, digestibility, ME	CP → microbial protein synthesis; ADF → limits digestibility; ME → energy availability	R^2^ ≈ 0.75–0.90; RMSE ≈ 0.15–0.25 kg DM m^−2^	[58,59]	Low ME → fatty liver; low digestibility → reduced milk yield
Satellite Optical/SAR Fusion (Sentinel-1 + Sentinel-2)	CP, NDF, DM, moisture	CP → microbial protein synthesis; NDF → intake potential; moisture → fermentation efficiency	R^2^ ≈ 0.65–0.85; RMSE ≈ 0.25–0.40 kg DM m^−2^	[58,59]	Low biomass → energy deficiency; moisture stress → ketosis
Sensor Fusion					
RPM + Spectral Calibration	CP, NDF, DM, WSC	CP → microbial protein synthesis; WSC → enhances microbial efficiency	R^2^ ≈ 0.70–0.88; RMSE ≈ 0.20–0.30 kg DM m^−2^	[59]	Low WSC → energy deficiency; CP/NDF imbalance → acidosis
RPM + OptRx (Optical Proximal Sensor)	CP	CP → nitrogen supply for microbial protein synthesis	R^2^ ≈ 0.74–0.86	[38]	Low CP → hypomagnesemia; high NDF → ruminal acidosis
ML/DSM/Trait Integration					
Machine Learning/AI	CP, NDF, DM, digestibility	CP → microbial protein synthesis; digestibility → energy utilization	R^2^ ≈ 0.70–0.90	[59]	Low CP → hypomagnesemia; low ME → fatty liver
Decision Support Models (DSM)	Biomass, CP, NDF, ME	CP → protein synthesis; ME → energy availability; NDF → intake potential	R^2^ ≈ 0.65–0.85	[58]	CP/NDF imbalance → acidosis; low ME → energy deficiency
Forage Trait Integration	CP, NDF, WSC, lignin, fatty acids	WSC → microbial efficiency; lignin → indigestible; fatty acids → modulate rumen fermentation	R^2^ ≈ 0.40–0.85	[60]	Low CP → hypomagnesemia; low WSC → ketosis; high lignin → digestive inefficiency

Ref—references; SSH—sward surface height; CSH—compressed sward height; DM—dry matter; GM—green matter; CP—crude protein; NDF—neutral detergent fiber; ADF—acid detergent fiber; WSC—water-soluble carbohydrates; ME—metabolizable energy; PMC—pasture moisture content. DSM—decision support models; ML/AI—machine learning/artificial intelligence; SARA—subacute ruminal acidosis.

## Data Availability

The original contributions presented in this study are included in the article. Further inquiries can be directed to the corresponding author.

## Abbreviations

The following abbreviations are used in this manuscript:

ADF	Acid Detergent Fiber
ADG	Average Daily Gain
AI	Artificial Intelligence
ALS	Aerial Laser Scanning
CNN	Convolutional Neural Network
CP	Crude Protein
DM	Dry Matter
DSS	Decision Support System
DSM	Decision Support Models
EE	Ether Extract
GPS	Global Positioning System
HSI	Hyperspectral Imaging
IoT	Internet of Things
LAI	Leaf Area Index
ME	Metabolizable Energy
MIR	Mid-Infrared Spectroscopy
ML	Machine Learning
NDF	Neutral Detergent Fiber
NDVI	Normalized Difference Vegetation Index
NIR	Near-Infrared
PA	Precision Agriculture
PAN	Precision Animal Nutrition
PLS	Personal Laser Scanners
PLF	Precision Livestock Farming
PS	Proximal Sensing
RFID	Radio-Frequency Identification
RS	Remote Sensing
SAR	Synthetic Aperture Radar
SARA	Subacute Ruminal Acidosis
SLS	Satellite Laser Scanning
TLS	Terrestrial Laser Scanning
UAV	Unmanned Aerial Vehicle
ULS	Uncrewed Aerial Laser Scanning
WOW	Walk-Over Weighting
WSC	Water-Soluble Carbohydrates
